# Duration of diaphragmatic inactivity after endotracheal intubation of critically ill patients

**DOI:** 10.1186/s13054-020-03435-y

**Published:** 2021-01-11

**Authors:** Michael Chaim Sklar, Fabiana Madotto, Annemijn Jonkman, Michela Rauseo, Ibrahim Soliman, L. Felipe Damiani, Irene Telias, Sebastian Dubo, Lu Chen, Nuttapol Rittayamai, Guang-Qiang Chen, Ewan C. Goligher, Martin Dres, Remi Coudroy, Tai Pham, Ricard M. Artigas, Jan O. Friedrich, Christer Sinderby, Leo Heunks, Laurent Brochard

**Affiliations:** 1 Keenan Research Centre, Li Ka Shing Knowledge Institute, St. Michael’s Hospital, 4th Floor, Room 411, 209 Victoria Street, Toronto, ON M5B 1T8 Canada; 2 Interdepartmental Division of Critical Care Medicine, University of Toronto, Toronto, Canada; 3 Value Based Health-Care Unit, IRCCS Multimedica, Sesto San Giovanni, Milan, Italy; 4 Department of Intensive Care Medicine, Amsterdam UMC, Location VUmc, Amsterdam, The Netherlands; 5 Departamento de Ciencias de La Salud, Facultad de Medicina, Pontificia Universidad Católica de Chile, Santiago, Chile; 6 Departamento de Kinesiologiá, Facultad de Medicina, Universidad de Concepción, Concepción, Chile; 7 Programa de Doctorado en Ciencias Médicas, Universidad de La Frontera, Temuco, Chile; 8 Division of Respiratory Diseases and Tuberculosis, Faculty of Medicine Siriraj Hospital, Mahidol University, Bangkok, 65106 Thailand; 9 Toronto General Hospital Research Institute, Toronto, ON Canada; 10 Division of Respirology, Department of Medicine, University Health Network and Sinai Health System, Toronto, ON Canada; 11 Pneumology and Critical Care Department, Public Assistance - Paris Hospital, Pitie-Salpetriere Hospital, Paris, France; 12 Médecine Intensive Réanimation, CHU de Poitiers, INSERM CIC1402 Alive Group, Université de Poitiers, Poitiers, France; 13 Service de Médecine Intensive-Réanimation, Hôpital de Bicêtre, Hôpitaux Universitaires Paris-Sud, Le Kremlin-Bicêtre, Paris, France; 14 Institute for Biomedical Engineering and Science Technology (iBEST), Ryerson University and St-Michael’s Hospital, Toronto, Canada

**Keywords:** Mechanical ventilation, Diaphragm, Electrical activity of the diaphragm, Sedation, Critical care

## Abstract

**Background:**

In patients intubated for mechanical ventilation, prolonged diaphragm inactivity could lead to weakness and poor outcome. Time to resume a minimal diaphragm activity may be related to sedation practice and patient severity.

**Methods:**

Prospective observational study in critically ill patients. Diaphragm electrical activity (EAdi) was continuously recorded after intubation looking for resumption of a minimal level of diaphragm activity (beginning of the first 24 h period with median EAdi > 7 µV, a threshold based on literature and correlations with diaphragm thickening fraction). Recordings were collected until full spontaneous breathing, extubation, death or 120 h. A 1 h waveform recording was collected daily to identify reverse triggering.

**Results:**

Seventy-five patients were enrolled and 69 analyzed (mean age ± standard deviation 63 ± 16 years). Reasons for ventilation were respiratory (55%), hemodynamic (19%) and neurologic (20%). Eight catheter disconnections occurred. The median time for resumption of EAdi was 22 h (interquartile range 0–50 h); 35/69 (51%) of patients resumed activity within 24 h while 4 had no recovery after 5 days. Late recovery was associated with use of sedative agents, cumulative doses of propofol and fentanyl, controlled ventilation and age (older patients receiving less sedation). Severity of illness, oxygenation, renal and hepatic function, reason for intubation were not associated with EAdi resumption. At least 20% of patients initiated EAdi with reverse triggering.

**Conclusion:**

Low levels of diaphragm electrical activity are common in the early course of mechanical ventilation: 50% of patients do not recover diaphragmatic activity within one day. Sedatives are the main factors accounting for this delay independently from lung or general severity.

*Trial Registration* ClinicalTrials.gov (NCT02434016). Registered on April 27, 2015. First patients enrolled June 2015.

## Introduction

In critically ill adults, diaphragm weakness is exceedingly common and has been described in more than 60% of patients [1]. There is mounting evidence that it may be associated with poor outcomes in mechanically ventilated patients, including difficult weaning, prolonged intensive care unit (ICU) stay, a higher risk of complications, and an increased mortality rate [2–5]. The most well-established form of mechanical ventilation related diaphragm injury (also known as myotrauma) is disuse atrophy, resulting from excessive ventilatory support, deep sedation and/or paralysis and has been referred to as over-assistance myotrauma [6, 7]. Daily examination of the diaphragm with ultrasound in critically ill ventilated patients has shown a progressive reduction of the thickness of the diaphragm occurring in the first three days in approximately 40% of patients [2, 3]. Studies in specific populations such as brain-dead organ donors have demonstrated muscle fiber atrophy and contractile dysfunction occurring within a few hours or days of controlled mechanical ventilation [8]. Diaphragm biopsies from ICU patients also demonstrated a reduction in active and passive diaphragm myofibrillar force and reduced cross-sectional area of slow-twitch and fast-twitch fibers as compared with control values [9–11]. Importantly, maintaining diaphragm activity under mechanical ventilation has been shown to be protective [12, 13]. Changes in diaphragm thickness were correlated with the amount of inactivity and are associated with complications of mechanical ventilation and a poor outcome [12, 13].

While the detrimental effects of diaphragm inactivity are widely recognized, no data yet exists regarding the duration of diaphragm inactivity after intubation in critically ill patients, and the factors associated with a delay in the resumption of minimal or normal diaphragm activity have not been explored. Such a delay may be primarily related to the severity of illness, the lung severity or the oxygenation defect, impacting the sedation delivered. Therefore, before considering implementing new strategies to mitigate the risks of diaphragm dysfunction in critically ill patients, the effects of current practices should be better understood. In this study, we aimed to assess the duration of absent or excessively low diaphragm activity after intubation by continuously measuring the electrical activity of the diaphragm (EAdi) soon after intubation in critically ill patients and also assessed the relationship between EAdi and diaphragm activity on ultrasound. We hypothesized that resumption of activity was influenced by disease severity and sedation management.

## Materials and methods

Full methods can be found in the Additional file 1.

### Study design

This prospective observational cohort study in acutely ill mechanically ventilated adult patients was approved by the Ethics Committee of St. Michael’s Hospital (REB #15-073) and registered on ClinicalTrials.gov (NCT02434016).

### Patients

To be enrolled, adult patients intubated in the ICU (or emergency department) were expected to have a duration of mechanical ventilation > 48 h (to exclude routine post-operative monitoring) and an oro- or nasogastric feeding tube equipped with electrodes at the level of the diaphragm (EAdi catheter, Getinge, Solna, Sweden), placed within 30 min after intubation (June 2015 to August 2017). Patients were excluded if there was anticipated removal of the catheter within 48 h of ICU admission for endoscopic interventions or need for magnetic resonance imaging (due to metal electrodes). Daily sedation interruption or minimization, and spontaneous breathing trials were performed as per the University of Toronto academic ICU policy [14].

### Experimental procedure

The EAdi catheter was positioned as described [15] using distance and with position confirmed based on the electrocardiogram tracings (the QRS amplitude decreases from top to bottom traces and the P wave disappears on the bottom tracing). Once inserted, the nasogastric tube was connected to a Servo-I® ventilator (Getinge, Solna, Sweden) equipped with a NAVA module (for EAdi recording). EAdi was then continuously recorded with trends captured every minute (value of the peak EAdi). Study recordings continued until EAdi was continuously above a threshold of 5–7 µV in a 24-h period assessed visually (see *outcome measures* for details), extubation, death or 120 h (5 days) of mechanical ventilation without achieving the primary outcome.

After ethics committee approval, an amendment to add in daily diaphragm ultrasound in consecutive patients, were performed over a few minutes to assess the relationship between EAdi and diaphragm thickening fraction (TFdi). Thickness of the diaphragm at end-expiration (Tdi,ee) and end-inspiration (TFdi,ei) was measured in M-mode as the distance between the parietal pleural and peritoneal membrane and TFdi was calculated as TFdi = (Tdi,ei − Tdi,ee)/Tdi,ee * 100%. A mean value of three breaths were taken and the average EAdi peak value corresponding to those breaths was recorded.

### Outcome measures and data collection

The primary outcome of interest was the time from intubation to resumption of EAdi > 7 µV for the next 24 h (see Additional file 1 for details and Additional file 2: Fig. E1). We decided to look for a sustained activity, defined as a median activity during a continuous 24-h period. The threshold of 7 µV corresponded to a minimal TFdi of 15%, which has been previously associated with a minimally acceptable inspiratory effort [2, 3], as per the correlation between EAdi and diaphragm activity on ultrasound in our subset of patients (see Results and Additional file 3: Fig. E2).

As we stopped recording EAdi after 120 h, the maximum onset time is 96 h from EAdi recording start. For patients with 120 h of EAdi recording and no diaphragm activity detected during this period, we assumed that the primary outcome occurred at a minimal time of 120 h. Secondary endpoints included the time to EAdi resumption using a threshold of 5 µV for a 24-h time window as a sensitivity analysis (see Additional file 1), and a 12-h time window, both for the 7 µV and 5 µV threshold.

A 1-h recording of ventilator and EAdi waveforms was also collected daily at a fixed time of the day to record and store EAdi–time, flow–time and pressure–time curves. The recording occurring within the 24-h period of EAdi resumption was reviewed and analyzed visually for the presence of spontaneous breathing efforts and reverse triggering dysynchrony [16]. Based on visual analysis, reverse triggering was defined by the presence of ventilator-initiated breaths (i.e. passive insufflation in controlled mechanical ventilation) followed by a patient spontaneous effort, i.e., indicating that the patient was triggered by the ventilator and not the reverse, usually with a repetitive pattern.

### Statistical methods

#### Sample size

About 40% of patients have a decrease in diaphragm thickness due to disuse within the first three days of mechanical ventilation [2]. Accordingly, having 70 patients would allow us to analyze approximately 30 patients with delayed or no resumption within the study period, and therefore obtain relatively precise estimates of the time to EAdi resumption and identifying late recovery of diaphragm activity. We added 5 subjects in case of dropout or technical problems related to EAdi recording.

#### Analysis

Baseline characteristics were summarized as means and standard deviations (SD), medians and interquartile ranges (IQR), or percentages. We performed two sets of analyses (see Additional file 1 for details):

1. *Time to EAdi resumption*: Survival analysis was performed to estimate the time to EAdi resumption, applying the Kaplan–Meier method accounting for any censoring that occurred before the fifth day of EAdi recording. As per this approach, the median time to EAdi resumption was calculated as the time point where 50% of participants experienced the event of interest. In addition, the mean (standard error) time to EAdi resumption was estimated as the area under the Kaplan Meier survival function curve. Note that the standard error was used as a measure of precision of this estimated mean.

In a univariate model, we analyzed main baseline and clinical characteristics associated with the time to EAdi resumption. Because the modality of ventilation (assisted or controlled) could change during the observation time, we used it as time-dependent covariate in the Cox model. The use and cumulative daily dose of continuous sedative infusions during the full study period were modelled as time-dependent covariates.

2. *Early vs. late resumption*: in a multivariable analysis, we compared early (< 24 h) vs late (≥ 24 h) resumption of EAdi accounting for variables present at baseline, until EAdi resumption in the early group or in the first 24 h for the late group. Differences between groups were assessed with the unpaired t-test or Mann–Whitney U test, according to the distribution. Categorical variables were compared with the use of the chi-square test or Fisher’s exact test, according to expected frequencies for each discrete variable. Logistic regression models were applied to investigate the relationship between main baseline and clinical characteristics and the probability to have a late resumption of EAdi, as well as the predictors for the use of sedative agents during the first 24 h (or until EAdi resumption if it occurred before 24 h).

*EAdi and TFdi*: The relationship between EAdi and TFdi was analyzed by a general linear regression model. Then, we classified data according to a TFdi value above or below 15% (i.e., minimal diaphragm contractile activity [3]); differences in EAdi between these groups were assessed with a Mann–Whitney *U* test.

All analyses were conducted using SAS version 9.4 (SAS Institute, Cary, NC, USA), R software version 3.3.2 (R Foundation for Statistical Computing, Vienna, Austria) and SPSS version 24.0 (IBM, Corp. USA). A *p* value less than 0.05 was considered to indicate statistical significance.

## Results

### Patient characteristics

**Fig. 1:**
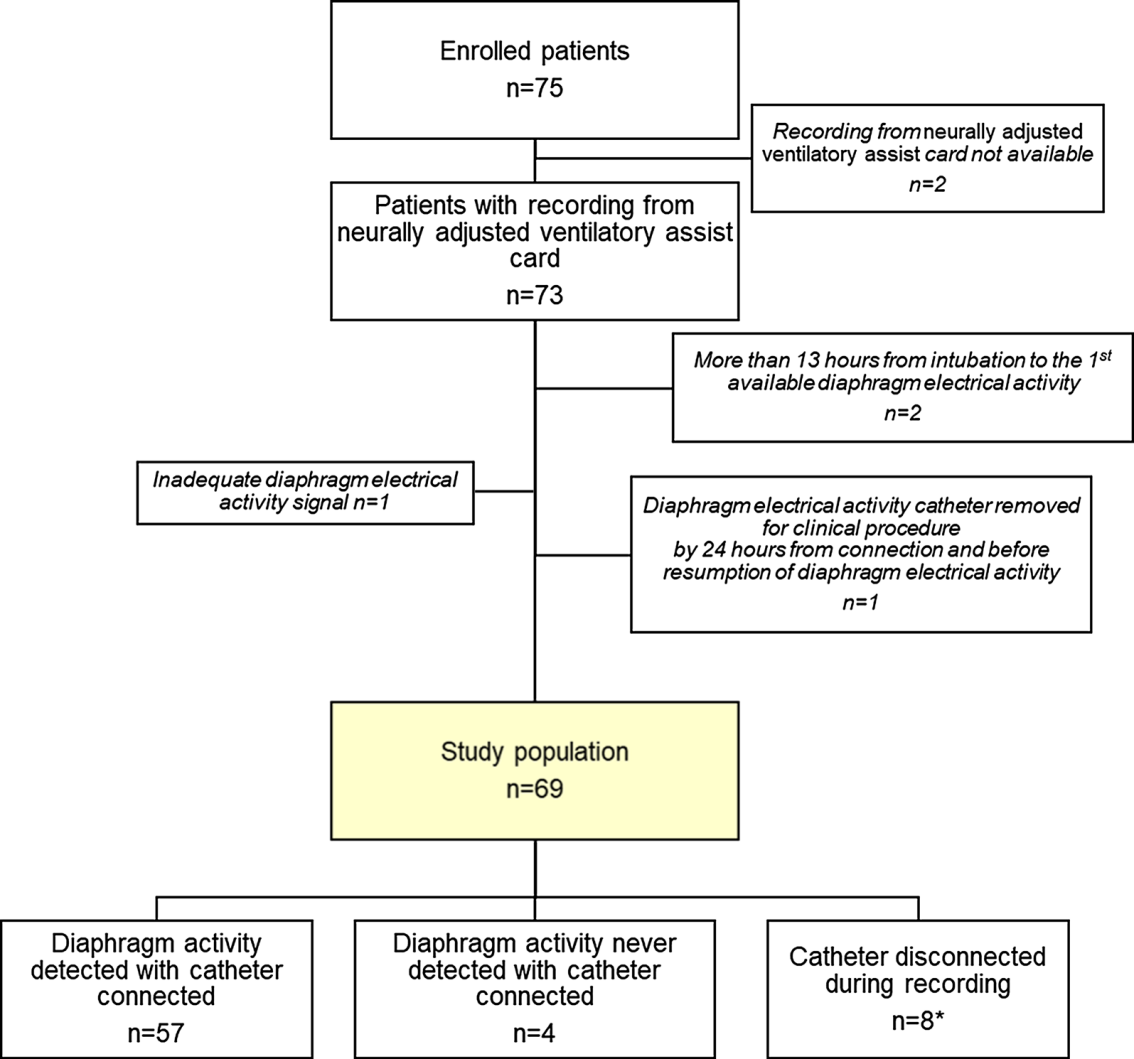
Flow chart of study population

Seventy-five patients were enrolled and 69 patients were kept in the analysis (Fig. 1). The characteristics at baseline are detailed in Table 1 and Additional file 1: Table E1. Most patients, 58/69 (84%) were critically ill medical patients and the reason for intubation was respiratory 38/69 (55%), hemodynamic 13/69 (19%) and neurologic 14/69 (20%) failure. Fifty-three (77%) patients received a bolus of neuromuscular blockade to facilitate endotracheal intubation. Following intubation and EAdi catheter placement, the initial mode of ventilation in 62/69 (90%) of patients was volume or pressure-assist-control ventilation and a spontaneous mode was used in 7/69 (10%) of patients as determined by the clinical team. In the first 24 h of EAdi recording, 63/69 patients (91%) received continuous sedative infusions and 4/69 patients (6%) received additional boluses or a continuous infusion of neuromuscular blockade. Among all patients’ characteristics, including gender, severity of illness scores, oxygenation, reason for intubation and kidney and liver function, the only factor associated with receiving a continuous sedative infusion in the first 24 h was younger age (odds ratio for age: 0.933, 95% CI 0.896–0.971, *p* = 0.0007) (Additional file 1: Table E2). The overall ICU and hospital mortality were 25% and 37% respectively. In the study period of 120 h, 90% of the cohort had continuous monitoring of EAdi, while for eight patients, unintended EAdi catheter disconnection happened prematurely (2 patients for palliation and 6 for catheter removal at time of clinical procedures such as magnetic resonance imaging) (Fig. 1 and Table 1).

**Table 1:** Characteristics of study population (*n* = 69)

Characteristic	Study population (*n* = 69)
Male, *n* (%)	41 (59.42)
Age (years), mean ± SD	63.33 ± 16.58
Body mass index, (kg/m^2^), mean ± SD	28.68 ± 9.31
Acute Physiologic Assessment and Chronic Health Evaluation (APACHE) II Scoring System (score)	
Mean ± SD	24.62 ± 8.28
Median (IQR)	24.50 [19.00–30.00]
Reason for ICU admission, *n* (%)	
Elective surgery	1 (1.45)
Emergency surgery	5 (7.25)
Trauma	5 (7.25)
Medical	58 (84.06)
Reason for intubation, *n* (%)	
Pulmonary	38 (55.07)
Hemodynamic	13 (18.84)
Neurologic	14 (20.29)
Airway protection	4 (5.80)
Intubation time, *n* (%)	
Day (6am–9 pm)	57 (82.61)
Night (9 pm–6am)	12 (17.39)
Arterial blood gas^a^	
FiO_2_, median (IQR)	0.50 [0.50–0.70]
PaO_2_ (mmHg), mean ± SD	121.84 ± 48.86
PaCO_2_ (mmHg), mean ± SD	44.16 ± 10.89
HCO_3_ (mmol/L), mean ± SD	23.58 ± 6.24
PaO_2_/FiO_2_ (mmHg), mean ± SD	226.36 ± 104.82
Baseline creatinine (µmol/L), median [IQR]	106.00 [71.00–176.00]
Baseline acute kidney injury stage, *n* (%)^f^	
No acute kidney injury	33 (47.83)
1	22 (31.88)
2	4 (5.80)
3	10 (14.49)
Baseline bilirubin (µmol/L)^c^, median [IQR]	10.00 [7.00–17.00]
Ventilatory settings at diaphragm electrical activity catheter connection	
Volume or pressure assist-control ventilation, *n* (%)	62 (89.86)
PEEP (cmH_2_O), mean ± SD	
All patients	8.05 ± 2.96
Patients on controlled mode (*n* = 62)	8.08 ± 3.08
Patients on assisted mode (*n* = 7)	7.83 ± 1.72
Mean airway pressure (cmH_2_O), mean ± SD	
All patients	12.50 ± 3.93
Patients on controlled mode (*n* = 62)	12.73 ± 4.01
Patients on assisted mode (*n* = 7)	10.54 ± 2.53
Time with available diaphragm electrical activity recording (hours), median (IQR)	69.20 [45.05–96.20]
Tracheostomy during ICU stay, *n* (%)^b^	7 (10.14)
Mortality, *n* (%)	
At ICU discharge	17 (24.64)
At hospital discharge^c^	25 (36.76)
Resumption of diaphragm electrical activity during study period^d^	
Subjects with complete recordings, *n* (%)	61 (88.41)
Time to resumption, *n* (%)	
< 24 h	35 (57.38)
24 h–48 h	11 (18.03)
48 h–72 h	6 (9.84)
72 h–96 h	5 (8.20)
> 96 h (no resumption)	4 (6.56)
Estimated time (hours) to resumption^e^	
Median (IQR)	22.02 [0.00–50.28]
Mean ± SE	35.20 ± 4.93
Average diaphragm electrical activity (µV) during resumption, mean ± SD	8.60 ± 7.07
Use of sedative during the first 24 h, *n* (%)^g^	63 (91.30)
Propofol, *n* (%)	49 (77.78)
Cumulative dose (mg/kg), median [IQR]	18.99 [9.60–32.86]
Midazolam, *n* (%)	46 (73.02)
Cumulative dose (mg/kg), median [IQR]	0.07 [0.03–0.59]
Fentanyl, *n* (%)	51 (80.95)
Cumulative dose (µg/kg), median [IQR]	4.40 [1.40–15.91]
Neuromuscular blocking agent use, *n* (%)^h^	
No	16 (23.19)
Yes	53 (76.81)
Yes, for intubation	49 (71.01)
Yes, additional bolus or continuous infusion	4 (5.80)
Sedation analgesia score (during the first 6 h of diaphragm electrical activity recording)^i^	
Median (IQR)	1.75 [1.00–2.50]

FiO_2_, fraction of inspired oxygen; HCO_3_, bicarbonate; ICU, intensive care unit; PaCO_2_, partial pressure of carbon dioxide; PaO_2_, partial pressure of oxygen; PEEP, positive end-expiratory pressure; SD, standard deviation; SE, standard error

^a^63 patients had ABG measured at baseline. For 1 patient HCO_3_ was not available

^b^Tracheostomy performed after 120 h from first available diaphragm electrical activity

^c^For 1 patient, data was missing

^d^Percentage was calculated on 61 patients with complete EAdi recordings

^e^Estimated time was assessed on whole study population (69 patients) with Kaplan–Meier approach. Time to resumption is calculated from first available diaphragm electrical activity monitoring using a threshold of 7 μV

^f^Acute kidney injury staging was determined using KDIGO Clinical Practice Guideline for Acute Kidney Injury

^g^6 patients did not receive continuous sedative infusions in the first 24 h. Note that patients may have received sedation after resumption of EAdi—see Additional file 1: Table E1

^h^Standard doses for neuromuscular blocking agents were used; intubation doses for rocuronium were 0.6–1.2 mg/kg, succinylcholine 1-2 mg/kg and cisatracurium 0.15–0.2 mg/kg. Continuous infusions of rocuronium or cisatracurium were titrated to clinical effect and were dosed as for 0.2 to 0.7 mg/kg/h and 0.06 to 0.18 mg/kg/h respectively as per hospital policy

^i^Sedation analgesia score not recorded in 54 patients in the first 6 h

### Time to EAdi resumption

**Fig. 2:**
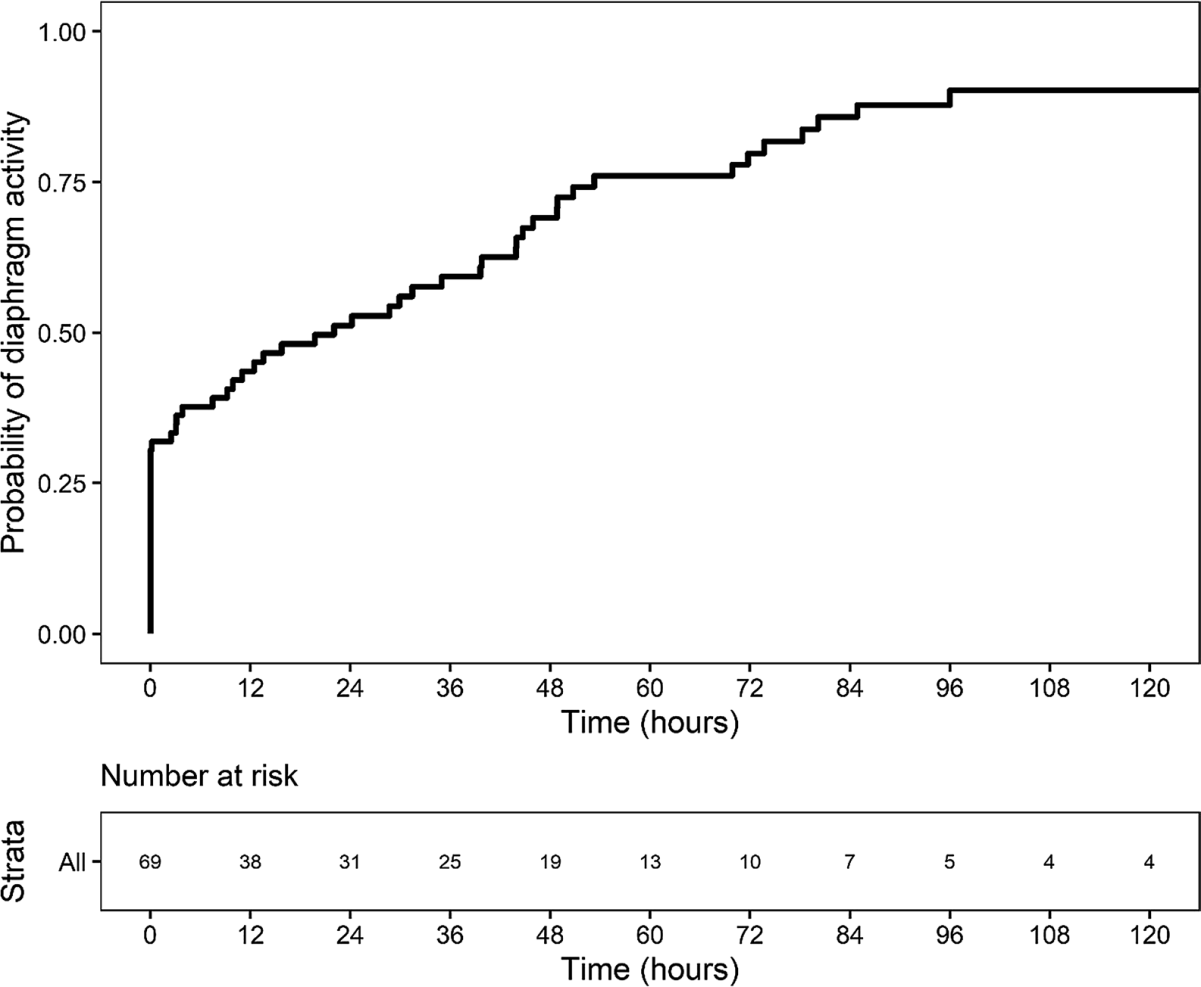
Probability to have EAdi resumption during the study period (*n* = 69). Following intubation, the estimated mean time for resumption of a minimal EAdi was 35 h (standard error 5 h) and the estimated median (IQR) time was 22 (0–50) h. *Note*: probabilities were estimated with the Kaplan–Meier approach and patients with catheter disconnection were censored at time of EAdi catheter disconnection

The estimated median (IQR) time for resumption of a minimal EAdi as defined per the primary endpoint was 22 (0–50) h and the estimated mean time was 35 h (standard error 5 h) for the study population (Table 1, Fig. 2). In total, 35/69 (51%) of patients had resumption of activity within 24 h, whereas 4/69 (6%) of patients had no EAdi resumption after 5 days (Table 1). Additional file 2: Fig. E1 shows the example of a recording with 4 days of total time of EAdi recording and who recovered EAdi approximately 48 h after intubation. The mean ± SD EAdi during the first 24 h of resumption was 8.60 ± 7.07 µV.

### Predictors of EAdi resumption

The infusion of at least one continuous sedative agent during the first 24 h (or until EAdi resumption if time to resumption was < 24 h) after intubation was associated with a significantly longer time to EAdi resumption (i.e., lower chance of resumption) with a hazard ratio of 0.18 (95% CI 0.10–0.32, *p* value: < 0.0001), and a time-dependent effect of dosing for propofol and fentanyl (*p* = 0.0029 and *p* = 0.0125, respectively). Older age was significantly associated with time to EAdi resumption (hazard ratio for age: 1.03, 95% CI 1.01–1.05, *p* = 0.0022). Time to EAdi resumption was not associated with the PaO2/FiO2 ratio at baseline, mean airway pressure, reason for intubation, time of intubation (day/night) or severity of illness scores in any analysis.

**Fig. 3:**
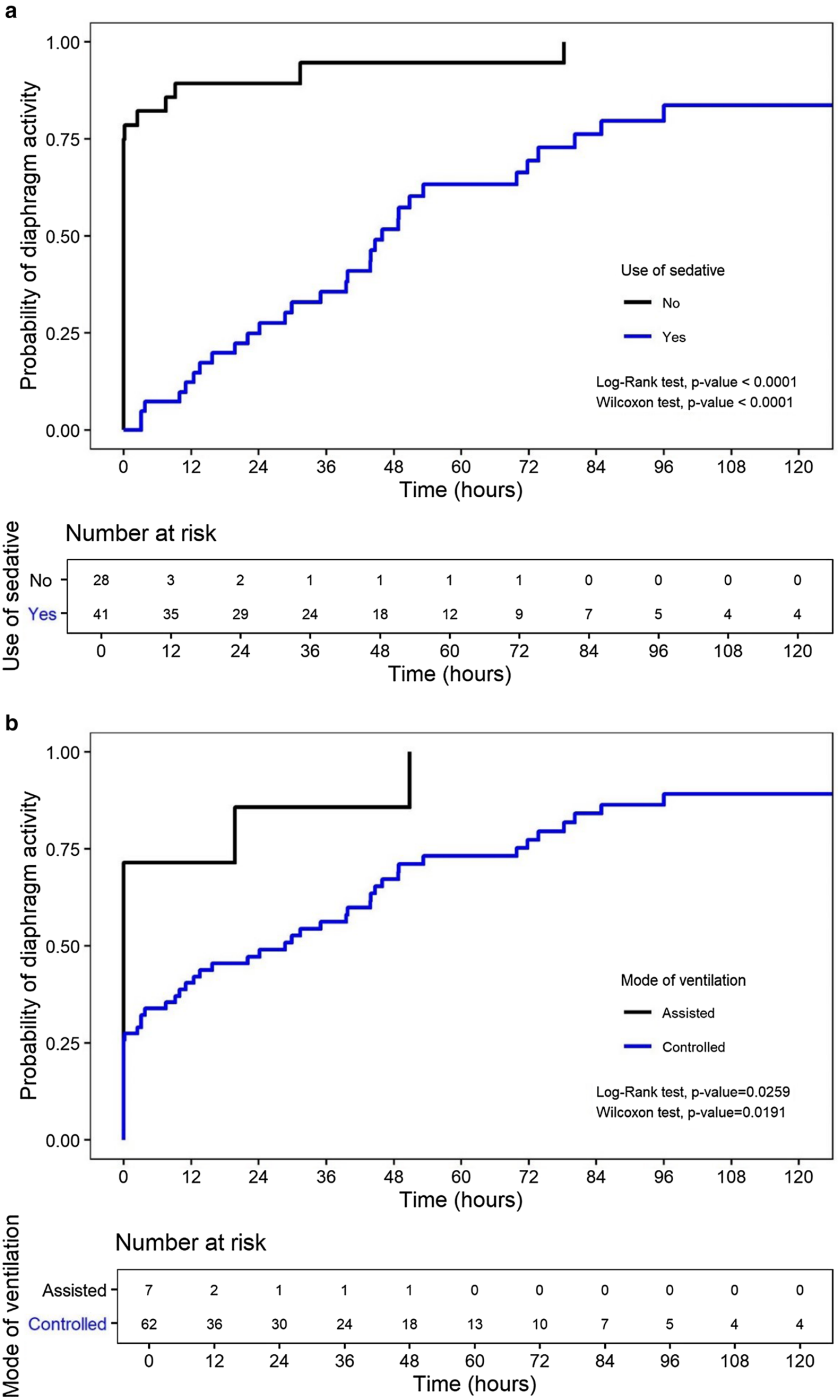
Probability of EAdi resumption during the study period stratified according to: (1) the use of sedatives (**a**); (2) mode of ventilation at EAdi catheter connection (assisted, controlled) (**b**). *Note*: probabilities were estimated with the Kaplan–Meier approach and patients with catheter disconnection were censored at time of EAdi catheter disconnection

**Fig. 4:**
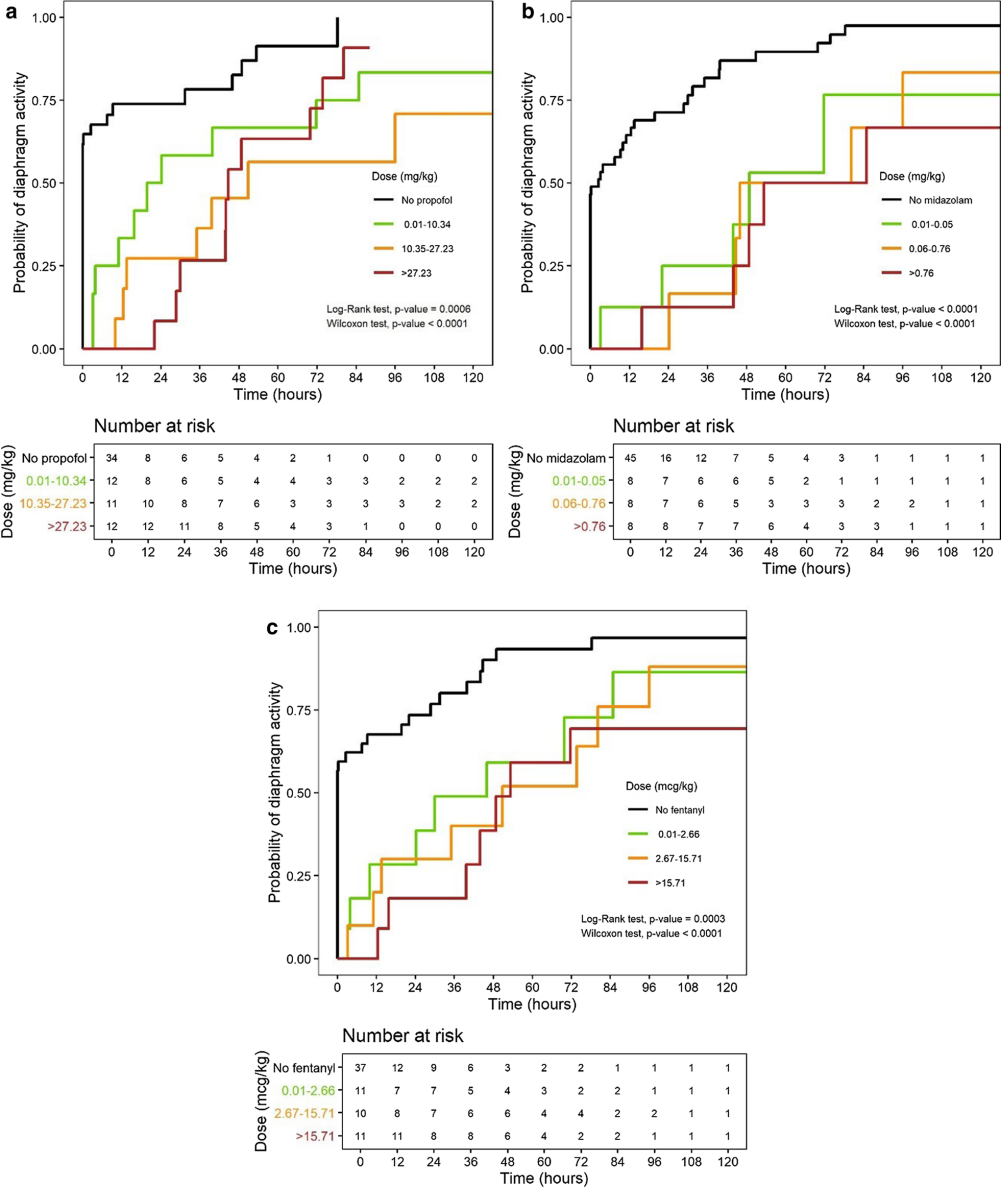
Probability of EAdi resumption during the study period stratified according to sedative drug: **a** propofol, **b** midazolam, **c** fentanyl

Figure 3a shows the probability of EAdi resumption during the study period in patients stratified according to the use of sedatives during the first 24 h (or until EAdi resumption if time to resumption was < 24 h). In the multivariable analysis, only the use of sedatives was associated with time to EAdi resumption, while the use of paralytic or sedation scores were not (Table 2). Although the mode of ventilation was not significantly associated with the time to EAdi resumption during the study period in the univariate logistic model (hazard ratio: 1.79, 95% CI 0.98–3.26, *p* = 0.0587), Fig. 3b shows that the probability of EAdi resumption was higher in patients on assisted modes of ventilation at the time of EAdi catheter connection compared to patients on controlled modes of ventilation (*p* = 0.0259 and *p* = 0.0191 for the Log-Rank and Wilcoxon test, respectively). Figure 4 shows the probability of EAdi resumption during the study period stratified according to sedative drug.

**Table 2:** Relationship between baseline parameters and EAdi resumption during the study period

	Hazard ratio (95% confidence interval)	*p* value	Patients
Univariate logistic models			
Age (year)	1.03 (1.01–1.05)	0.0022	69
Sex (ref. Female)	0.97 (0.58–1.63)	0.9179	69
Acute Physiologic Assessment and Chronic Health Evaluation (APACHE) II Scoring System (score)	1.02 (0.98–1.06)	0.2900	68
Body mass index, (kg/m^2^)	1.02 (0.99–1.05)	0.1771	67
Mode of ventilation (ref. assisted)	1.79 (0.98–3.26)	0.0587	69
PaO_2_/FiO_2_ (mmHg)	1.00 (1.00–1.00)	0.3537	63
Creatinine (µmol/L)	1.00 (1.00–1.00)	0.2590	69
Baseline acute kidney injury stage (ref. No acute kidney injury)			69
1	1.76 (0.96–3.20)	0.0665	
2	0.87 (0.26–2.89)	0.8155	
3	1.64 (0.75–3.62)	0.2178	
Baseline bilirubin (µmol/L)	1.00 (0.99–1.01)	0.8071	68
Cause of intubation (ref. Neurologic)			65*
Pulmonary	1.14 (0.60–2.18)	0.6904	
Hemodynamic	0.93 (0.40–2.13)	0.8578	
Sedation analgesia score during the first 6 h (score)	1.19 (0.88–1.60)	0.2639	54
Use of sedative (ref. No)	0.18 (0.10–0.32)	< .0001	69
Use of sedative (ref. No)—time dependent	0.09 (0.04–0.21)	< .0001	69
Use of Propofol (ref. No)	0.34 (0.19–0.60)	0.0002	69
Use of Propofol (ref. No)—time dependent	0.32 (0.18–0.57)	0.0001	69
Dose propofol (1 mg/kg)	0.98 (0.96–1.00)	0.0334	69
Dose propofol (1 mg/kg)—time dependent	0.98 (0.96–0.99)	0.0029	69
Use of Midazolam (ref. No)	0.29 (0.16–0.52)	< .0001	69
Use of Midazolam (ref. No)—time dependent	0.29 (0.15–0.55)	0.0002	69
Dose midazolam (0.01 mg/kg)	1.00 (0.99–1.00)	0.1018	69
Dose midazolam (0.01 mg/kg)—time dependent	0.96 (0.91–1.00)	0.0603	69
Use of Fentanyl (ref. No)	0.36 (0.21–0.62)	0.0002	69
Use of Fentanyl (ref. No)—time dependent	0.36 (0.20–0.64)	0.0004	69
Dose fentanyl (1 mcg/kg)	0.99 (0.97–1.00)	0.1540	69
Dose fentanyl (1 mcg/kg)—time dependent	0.98 (0.96–0.99)	0.0125	69
Neuromuscular blocking agent use (ref. No)	0.78 (0.43–1.43)	0.4262	69
Multivariable model with time dependent variables			
Model 1			
Use of sedative (ref. No)—time dependent	0.09 (0.04–0.21)	< .0001	69
Model 2			
Use of Midazolam (ref. No)—time dependent			
Without propofol	0.49 (0.22–1.10)	0.0799	
With propofol	0.11 (0.05–0.26)	< .0001	
Multivariable model (no time dependent variables)			
Model 1			
Use of sedative (ref. No)	0.18 (0.10–0.32)	< .0001	69
Model 2			69
Use of Midazolam (ref. No)	0.31 (0.17–0.566)	0.0001	
Use of Propofol (ref. No)	0.38 (0.21–0.67)	0.0009

### Early vs Late EAdi resumption

When patients with early EAdi resumption (< 24 h) were compared to patients with late EAdi resumption (≥ 24 h), no statistical relationship was found for sex, APACHE-II score, body mass index, renal or hepatic function, reason for intubation, positive end-expiratory pressure, mean airway pressure (as a surrogate of the intensity of ventilation) or arterial blood gas parameters at baseline. Patients with early EAdi resumption were older (68.6 ± 15.9 vs 57.9 ± 15.7 for early vs. late EAdi resumption, *p* = 0.0043). Details on the study population stratified by early (< 24 h, 51%) vs late (≥ 24 h, 49%) EAdi resumption are reported in Additional file 1: Table E1. Continuous sedation after intubation and increased doses of propofol and fentanyl were the only factors associated with a late resumption (Table 3).

**Table 3:** Relationship between baseline parameters and probability to have a late resumption of diaphragm electrical activity (after 24 h) during the study period

	Odds ratio (95% confidence interval)	*p* value	*N*
Univariate logistic models			
Age (year)	0.958 (0.928–0.990)	0.0101	69
Sex (ref. Female)	0.748 (0.286–1.962)	0.5557	69
Acute Physiologic Assessment and Chronic Health Evaluation (APACHE) II Scoring System (score)	0.975 (0.919–1.034)	0.3949	68
Body mass index, (kg/m^2^)	0.987 (0.936–1.040)	0.6146	67
Mode of ventilation (ref. assisted)	6.824 (0.776–60.040)	0.0835	69
PaO_2_/FiO_2_ (mmHg)	1.002 (0.997–1.007)	0.3904	63
Creatinine (µmol/L)	1.002 (0.998–1.005)	0.4109	69
Baseline acute kidney injury stage (ref. no acute kidney injury)			69
1	0.439 (0.142–1.356)	0.1526	
2	2.824 (0.266–30.021)	0.3894	
3	2.196 (0.483–9.991)	0.3088	
Baseline bilirubin (µmol/L)	1.005 (0.988–1.022)	0.5666	68
Cause of intubation (ref. Neurologic)			65*
Pulmonary	0.324 (0.086–1.217)	0.4076	
Hemodynamic	0.250 (0.050–1.251)	0.2145	
Sedation analgesia score during the first 6 h (score)	0.732 (0.408–1.313)	0.2950	54
Use of sedative (ref. No)	25.833 (6.411–104.094)	< .0001	69
Use of propofol (ref. No)	6.944 (2.412–19.994)	0.0003	69
Dose of propofol (1 mg/kg)	1.090 (1.031–1.151)	0.0022	69
Use of midazolam (ref. No)	17.231 (4.375–67.860)	< .0001	69
Dose of midazolam (0.01 mg/kg)	1.026 (0.998–1.055)	0.0666	69
Use of fentanyl (ref. No)	8.100 (2.750- 23.855)	0.0001	69
Dose of fentanyl (1 mcg/kg)	1.073 (1.003–1.148)	0.0394	69
Multivariable logistic model			69
Neuromuscular blocking agent use (ref. No)	2.658 (0.811–8.713)	0.1067	69

^*^4 patients with other causes of intubation were excluded

### Relationship between EAdi and TFdi

The correlation between EAdi and TFdi was performed in twenty-one patients with adequate ultrasound measurements available. We excluded one patient with major chronic lung distension and a flat diaphragm, making the reliability of thickening fraction measurements questionable. The correlation is shown in Additional file 3: Fig. E2a and could be described as TFdi (%) = 6.18 + 1.25*EAdi (*R*^2^ = 0.623, *p* < 0.001). A minimal TFdi of 15% as previously defined would thus correspond to EAdi > 7 µV. The median (IQR) EAdi during the ultrasound recording was 2.06 (0.53–6.5) µV and 14.75 (7.2–29) µV for those patients with TFdi below and above 15% respectively (Additional file 3: Fig. E2b, *p* = 0.001 for differences between groups).

### Different definitions for EAdi resumption

Using a threshold of 7 µV and 12-h time period, the estimated time for resumption of a minimal EAdi was median (IQR) 18.40 (0.00–53.88) hours. Using a threshold of 5 µV, the time for resumption of EAdi for a 24-h and 12-h time period were 22.02 (0.00–50.77) h and 18.38 (0.00–53.88) h , respectively (Additional file 1: Table E3).

### Presence of reverse triggering at time of resumption of EAdi

In 55 patients with a 1-h recording available within the first 24 h of EAdi resumption, ventilator and EAdi waveforms were analyzed: 47 patients showed spontaneous efforts triggering the ventilator, 6 patients had evidence of reverse triggering with no spontaneous effort triggering the ventilator and 2 patients had a mixture of reverse triggering and spontaneous efforts over this hour. For patients who were already triggering the ventilator during this 1-h recording, we reviewed tracings from the preceding day when they were on a controlled mode of ventilation and 3 additional patients showed reverse triggering (total of 11/55 patients, 20%). Considering only the 29 patients under controlled ventilation either the day before or the day of EAdi resumption, reverse triggering was present in 38% of cases.

## Discussion

In this short-term observational physiological study, we evaluated the temporal pattern of EAdi resumption in critically ill adult patients requiring endotracheal intubation. The prevalence of excessively low diaphragm activity in the first hours after intubation was high and the median time to EAdi resumption was 22 h. Younger patients and those on higher doses of sedative infusions in the first 24 h of mechanical ventilation experienced delayed resumption of EAdi. Delayed EAdi resumption was not associated with the reason for intubation, the severity of illness, renal or hepatic dysfunction, the level of intensity of ventilation or oxygenation defect. Around the time of EAdi resumption, at least 20% of patients exhibited reverse triggering and this was the case for almost 40% of those still on controlled ventilation. EAdi amplitude was well correlated with TFdi as measured with ultrasound.

Our results show that an absent or abnormally low diaphragm activity is present in almost half of the patients (49%) for more than 24 h after intubation. Decreases in diaphragm strength and fiber cross-sectional area that have been demonstrated in both animal and human studies [2, 17–19] might thus be a consequence of frequently suppressed activity early during invasive mechanical ventilation.

When clinically stable, transitioning a patient to spontaneous breathing using assisted modes of ventilation with reduced or interrupted sedation might preserve diaphragm activity and prevent the development of disuse atrophy. This approach, however, must be weighed against the need for lung protection [7], especially in the presence of excessive respiratory drive. We confirmed that a transition to assisted modes of ventilation, however, does not imply direct resumption of normal EAdi levels. In fact, the relatively low EAdi values in our patients might be the result of ventilator over-assistance, which is common and often unnoticed in modes such as pressure support ventilation [2, 20, 21]. This underscores the potential importance of monitoring respiratory effort during mechanical ventilation in order to target diaphragm activity within a safe physiological range [3, 7].

Our analysis shows that the presence of a continuous sedative infusion after intubation is the main risk factor for delayed EAdi resumption, while severity of illness, including intensity of ventilation and oxygenation, renal and hepatic function, reason for intubation and other clinical factors were not statistically associated. Our findings support a previous single center randomized controlled trial of a “no sedation” approach in mechanically ventilated patients who had significantly reduced time on the ventilator [22, 23]. Our findings confirm that the prescription of continuous sedation has a major impact on the resumption of diaphragm activity and this seems independent from a patient’s clinical severity. Although the effect of sedation was expected, the lack of relationship with severity suggests that the administration of sedation may not be dictated by the needs of the patients but more by predetermined behavior, habits and/or protocols. Also, these findings suggest that the administration of sedation may be amenable to improvement. Somewhat unexpectedly, we found that older patients had an earlier resumption of EAdi compared to younger ones. This finding is explained by the fact that older patients also received significantly less propofol, suggesting that clinicians limit continuous sedation in older patients given the potential for exacerbating delirium or risk of deep sedation. This also confirms indirectly that sedation might be administered based on clinicians’ decisions. Although our study did not capture in more granular details the characteristics of the patient at time of early ventilation, this result suggests that sedation administration for maintaining respiratory muscle activity could be better controlled.

A median EAdi threshold of 7 µV for 24 h was chosen to represent a resumption of clinically relevant EAdi. In our initial protocol we were interested in the first time EAdi values were > 5 µV, but there was significant variability in the EAdi signals and a “one-time” value of 5 µV for a few minutes was often very transient, (Additional file 4: Fig. E3), with a high risk of noise [24]. This voltage threshold value was chosen arbitrarily as half of the minimal 10 µV value observed in different studies, and it was reasoned that this would represent a conservative value for resumption of minimal EAdi. Healthy subjects have median EAdi values during normal spontaneous breathing close to 17 µV and above 10 µV in 92% of subjects [25]. We found a significant correlation between EAdi and TFdi, which demonstrated that a minimal TFdi of 15% as previously defined [2, 3], would correspond to an EAdi > 7 µV. This physiological rationale justified using a threshold of 7 µV, while performing a sensitivity analysis using a threshold of 5 µV, with essentially identical results, as well as analyses performed at an onset time of 12-h and 24-h, again with very similar results.

The presence of patient-ventilator dysynchrony may be driven by complex interactions between sedatives and opioids [26]. We were particularly interested in reverse triggering [16], which was frequently observed at the time minimal EAdi was detected during controlled mechanical ventilation. The physiological mechanism and clinical consequences of this specific dysynchrony are subject of ongoing research. Reverse triggering can lead to breath-stacking and might be associated with lung and diaphragm injury. In contrast, reverse triggering may also be a protective reflex to prevent diaphragm atrophy and further investigation is required to better understand its consequences. Although our analysis was limited by having only a 1 h recording at a fixed time of the day, therefore underestimating the true incidence, we observed that reverse triggering may be a frequent way for diaphragm activity to restart. This is further supported by previous research which demonstrated an increased amount of dysynchrony with higher as compared to lower dose propofol for sedation [27].

To improve ventilatory management of the critically ill patient our data suggest that monitoring of respiratory drive and inspiratory effort could be helpful [28, 29]. Low respiratory drive and effort as a result of ventilator over-assistance and/or excessive sedation may lead to diaphragm atrophy and dysfunction, while strong inspiratory efforts might lead to load-induced diaphragm injury [3, 6, 28]. Clinicians frequently give sedation to treat or even prevent obvious patient-ventilator dysynchrony, but this is probably at the expense of excessive sedation. EAdi amplitudes vary between individuals and safe upper limits for diaphragm activity are currently unknown. A diaphragm-protective ventilation strategy might employ EAdi monitoring or other techniques to identify patients at risk for ventilator over-assistance myotrauma, for assessing changes in respiratory drive, and to adjust ventilator settings accordingly [25]. EAdi is not a direct measure of breathing effort but studies have shown reasonable correlation with functional parameters such as muscular pressure (Pmus) [30]. Here we demonstrate a reasonably strong correlation between EAdi and diaphragm thickening measured by ultrasound in critically ill mechanically ventilated patients. A tidal variation in TFdi in the range of 15 to 30% was associated with shorter duration of mechanical ventilation in a large cohort of critically ill patients [3]. Accordingly, TFdi below 15% might put the patient at risk for development of disuse atrophy [3, 31]. The current study shows that patients with TFdi values below 15% indeed had abnormally low EAdi amplitudes, which was significantly different from patients with normal TFdi and strengthens the rationale for titrating ventilator assistance to reach a minimal TFdi of 15%.

## Limitations

This study had a limited number of enrolled subjects and was conducted in a single ICU. Since we found sedation practice to be a significant contributor, it is possible that location and practice variation could influence the time and magnitude of EAdi resumption. However, sedation practices and therefore EAdi resumption were not associated with patient severity of illness or oxygenation deficit suggesting that this practice may be modified. Pain, agitation, delirium and sleep are complex pathophysiological processes in critically ill patients [32]. Clinically, each of these components should be addressed separately and assuming that all intravenous agents act uniformly on EAdi recovery is an oversimplification. As analgesics, opioids seem to have vastly different effects on respiratory function and muscle activity compared to other agents, manifested in reductions in ventilator asynchrony and less suppressed EAdi activity compared to non-opioid based strategies [26, 27]. Conversely, propofol based sedation, even in healthy volunteers, has a profound impact on diaphragm activity [33]. While we attempted to separately analyze individual agents, they were used most often in combination. Future research in this area should attempt to study the individual effects of agents more precisely.

Secondly, we enrolled 75 patients in a 2-year time frame, which is much less than the number of acutely intubated patients in our ICU. This was explained in part by the logistical difficulty to conduct such a study in which recordings had to start almost at time of intubation. Our findings have reasonable external validity as the baseline characteristics of our cohort were representative of a general medical ICU patient population, but the actual number of surgical patients was low limiting our ability to draw associations in that cohort. Third, we felt that we used a reasonable threshold to detect minimal EAdi activity, as confirmed with our ultrasound data, but did not measure directly the effort of the patient. Although it would have been less sensitive, this could also be assessed with transdiaphragmatic pressure measurements, but would have added to the complexity and time sensitive nature of the measurements. Moreover, we focussed only on diaphragm activity in this study, while the time to recovery of other inspiratory muscle activity may be interesting to characterize. Finally, there were episodes of intermittent catheter dislodgement which required adjustment of positioning and in 7 patients the inability to measure recordings for the complete duration of the study. These catheter disconnections occurred after 24 h and the patients were kept in the early vs late comparison.

## Conclusion

Following endotracheal intubation, critically ill adults experience suppression of diaphragm activity and the time to resumption of a reasonably minimal EAdi takes almost one day. Continuous infusions of sedatives and use of controlled modes of mechanical ventilation contribute to delayed recovery while severity of illness scores did not. Our results suggest that sedation practices in particular contribute to prolonged disuse of the respiratory muscles with a risk of diaphragm atrophy independent of patient severity of illness. Better monitoring of respiratory drive and integration of breathing effort to sedation practice algorithm may be of benefit.

## Supplementary Information

**Additional file 1.** Methods, Results and Tables.

**Additional file 2.** Figure E1.

**Additional file 3.** Figure E2.

**Additional file 4.** Figure E3.

## Data Availability

The datasets used and/or analysed during the current study are available from the corresponding author on reasonable request.

## Abbreviations

EAdi: Electrical activity of the diaphragm
ICU: Intensive care unit
TFdi: Thickening fraction of the diaphragm
