# Supplementary material for: Duration of diaphragmatic inactivity after endotracheal intubation of critically ill patients
Source: Crit Care. 2021 Jan 11;25:26. doi: 10.1186/s13054-020-03435-y (PMC7798017; doi:10.1186/s13054-020-03435-y)
Supplement: Supplementary file 1 — Additional file 1. Methods, Results and Tables. [file 13054_2020_3435_MOESM1_ESM.docx]

**Duration of diaphragmatic inactivity after endotracheal intubation of critically ill patients**

Michael. C. Sklar, Fabiana Madotto, Annemijn Jonkman, Michela Rauseo, Ibrahim Soliman, L. Felipe Damiani, Irene Telias, Sebastian Dubo, Lu Chen, Nuttapol Rittayamai, Gong.-Qiang. Chen, Ewan C. Goligher, Martin Dres, Remi Coudroy, Tai Pham, Ricard M. Artigas, Jan O. Friedrich, Christer Sinderby, Leo Heunks, Laurent Brochard

Electronic supplement

**Materials and Methods**

Full methods can be found in the electronic supplement

*Study design*

This prospective observational cohort study in acutely ill mechanically ventilated adult patients was approved by the Ethics Committee of St. Michael’s Hospital (REB #15-073) and registered on ClinicalTrials.gov (NCT02434016). Patients were included by waiver of consent since the use of feeding tubes allowing EAdi monitoring was standard in the ICU.

*Patients*

To be enrolled, adult patients intubated in the ICU (or emergency department) were expected to have a duration of mechanical ventilation > 48 hours (to exclude patients admitted to the ICU for routine post-operative monitoring) and an oro- or nasogastric feeding tube equipped with electrodes at the level of the diaphragm (EAdi catheter, Getinge, Solna, Sweden), placed within 30 minutes after intubation between June 2015 and August 2017. Patients were excluded if there was anticipated removal of the catheter within 48 hours of ICU admission for endoscopic interventions or presumed need for magnetic resonance imaging (due to metal electrodes). Patients were also excluded if there was a history of phrenic nerve lesions, high risk for intracranial hypertension, proven intracranial hypertension (as discontinuation of sedation would be potentially dangerous in this group), or those who were already intubated for at least 12 hours. Patients were managed as per the attending physician and clinical team. When appropriate, daily sedation interruption or minimization, and spontaneous breathing trials were performed as per the University of Toronto academic ICU policy[1].

*Experimental procedure*
The EAdi catheter was positioned as previously described [2] carefully using distance and with position confirmed based on the electrocardiogram tracings (the QRS amplitude decreases from top to bottom traces and the P wave disappears on the bottom tracing, indicating that the last electrode is below the diaphragm). Once inserted, the nasogastric tube was connected to a Servo-I® ventilator (Getinge, Solna, Sweden) equipped with a NAVA module (for EAdi recording). EAdi was then continuously recorded with trends captured every minute (value of the peak EAdi). Study recordings continued until EAdi was continuously above a threshold of 5-7 µV in a 24-hour period assessed visually (see *outcome measure* for details), extubation, death or 120 hours (5 days) of mechanical ventilation without achieving the primary outcome.

In some of the patients, daily diaphragm ultrasound measurements were also performed over a few minutes to assess the relationship between EAdi and diaphragm thickening fraction (TFdi). Ultrasound images were acquired from the right hemi-diaphragm as visualized in the zone of apposition using a high frequency (10-15 MHz) linear array transducer (FUJIFILM Sonosite) as previously described [3]. Thickness of the diaphragm at end-expiration (Tdi,ee) and end-inspiration (TFdi,ei) was measured in M-mode as the distance between the parietal pleural and peritoneal membrane and TFdi was calculated as TFdi = (Tdi,ei – Tdi,ee) / Tdi,ee *100%. A mean value of three breaths were taken and the average EAdi peak value corresponding to those breaths was recorded.

*Outcome measures and data collection*
The primary outcome of interest was the time from intubation to resumption of EAdi. In our initial protocol, we wanted to look at the first time EAdi was above 5 µV even for a very brief period. There was no consensus in the literature to agree on what is a normal EAdi. This initial EAdi threshold was based on data in healthy subjects showing that normal values are usually above 10 µV [4], the average values of data in published studies in adults showing values of approximately 10 µV [5], and a systematic review in pediatric patients showing that the average EAdi values in patients under NAVA were approximately 10 µV[6]. We reasoned that choosing a low level would be conservative for describing abnormally low diaphragm activity and sensitive to detect an early start of moderate electrical activity. Before performing the final analysis, we later decided to make two changes in our statistical plan: first, after examining the waveforms of 25% of the patients, we realized that brief and frequent fluctuations in activity made such a brief endpoint uninterpretable. We then decided to look for a more clinically relevant end-point, and to look for a sustained activity, defined as a median activity during a continuous 24-hour period: the time of resumption was therefore the (first) time at which the median EAdi was > 5 µV during the next continuous 24 hours (**Figure e1).** Second, we looked at the correlation between EAdi and diaphragm activity on ultrasound in our subset of patients, obtained once per day: we found that a TFdi of 15% corresponded approximately to 7 µV of EAdi (see Results). We therefore decided to change our primary endpoint to the first time at which the median EAdi is >7 µV for the next 24 hours, and to keep our initial threshold of 5 µV as a sensitivity analysis.

As we stopped recording EAdi after 120h, the maximum onset time is 96 hours from EAdi recording start. For patients with 120 hours of EAdi recording and no diaphragm activity detected during this period, we assumed that the primary outcome occurred at a minimal time of 120 hours. Secondary endpoints included the time to EAdi resumption using only a 12-hour time window, both for the 7 µV and 5 µV threshold.

Members of the study team were on call to enroll patients and to conduct study measurements. The medical record was used to collect patient demographics, severity of illness scores, renal and hepatic function and clinical outcomes. Renal function was described as creatinine levels on admission and acute kidney injury stage at 24 hours based on KDIGO guidelines[7] and hepatic function was evaluated by serum total bilirubin values at 24 hours. The paper chart, nursing flow sheets and respiratory therapist records were used to collect the time of intubation and doses of continuous infusions of sedative agents. The depth of sedation was measured by the Riker scale [8] and ventilatory management strategies including arterial blood gases and ventilator settings were also recorded. Mean airway pressure was used as a marker of the intensity of ventilation and could be compared across both assisted and controlled modes of ventilation. In addition to the continuous trends recordings of EAdi (giving values every minute), a one-hour recording of ventilator and EAdi waveforms was also collected daily at a fixed time of the day: the Servo-I^®^ ventilator was connected through a RS232 cable to a laptop equipped with dedicated software (Servo-tracker^®^ software version 4.2, Getinge) to record and store EAdi–time, flow–time and pressure–time curves. This one-hour recording occurring within the 24-hour period of EAdi resumption was reviewed and analyzed visually for the presence of spontaneous breathing efforts and reverse triggering dysynchrony [9]. Based on visual analysis, reverse triggering was defined by the presence of ventilator-initiated breaths (i.e. passive insufflation in controlled mechanical ventilation) followed by a patient spontaneous effort, i.e., indicating that the patient was triggered by the ventilator and not the reverse, usually with a repetitive pattern.

*Statistical methods*

*Sample size*
Estimates of time from catheter positioning to EAdi resumption were not available in the scientific literature and the main aim of this exploratory study was to provide detailed clinical information on this parameter for future trials. A minimum convenient sample size of at least 25-30 patients was required for adequate exploration of the objective. Previous data using diaphragm ultrasound showed that about 40% of patients have a decrease in diaphragm thickness due to disuse within the first three days of mechanical ventilation[3]. Accordingly, having 70 patients would allow us to analyze approximately 30 patients with delayed or no resumption within the study period, and therefore obtain relatively precise estimates of the time to EAdi resumption and identifying late recovery of diaphragm activity. We added 5 subjects to the final sample size in case of dropout or technical problems related to EAdi recording.

*Analysis*

Baseline characteristics were summarized as means and standard deviations (SD), medians and interquartile ranges, or percentages. For the present study we performed two sets of analyses:

1. Time to EAdi resumption: in a univariate model, we analyzed factors associated with the time to EAdi resumption based on our primary endpoint and included time-dependent variables (if necessary). Survival analysis was performed to estimate the time to EAdi resumption, applying the Kaplan-Meier method accounting for any censoring that occurred before the fifth day of EAdi recording. In detail, the mean time was calculated as the area under the estimated survival curve, variability was assessed with Greenwood’s standard error formula and the median time was calculated as the time for which the survival function was 0.5 (i.e. determined as the time when half the subjects had EAdi resumption). The Kaplan-Meier method was also used to show and compare (Log-Rank and Wilcoxon test) the probability of having EAdi resumption during the study period (120 hours), in the study population stratified by the main baseline and clinical characteristics. To assess the association between each characteristic and the probability to have EAdi resumption during the study period, we performed Cox proportional-hazards models and results were reported as hazard ratio (HR) and 95% confidence interval (CI). A stepwise approach was applied to detect the set of independent variables significantly associated to the outcome. This approach combines forward and backward selection methods (with a significance level of 0.05 both for entry and retention) in an iterative procedure to select predictors in the final multivariable model. The validity of the proportional hazards assumption in the Cox regression models was assessed with the test proposed by Harrel and Lee based on Schoenfeld residuals and Kolmogorov-type supremum test for continuous covariates [10,11]. Martingale residuals of null Cox regression models were used to check the assumption of linearity in the relationship between the log hazard and continuous covariates. Because the modality of ventilation (assisted or controlled) could change during the observation time, we used it as time-dependent covariate in the Cox model. The use and cumulative daily dose of continuous sedative infusions during the full study period were modelled as time-dependent covariates.

2. Early vs. late resumption: in a multivariable analysis, we also compared early (24h) vs late resumption of EAdi accounting for variables present at baseline, until EAdi resumption in the early group or in the first 24 hours for the late group. Differences between groups in baseline and clinical characteristics were assessed with the unpaired t-test or Mann-Whitney U test, according to the distribution of each continuous variable. Categorical variables were compared with the use of the chi-square test or Fisher’s exact test, according to expected frequencies for each discrete variable. Moreover, logistic regression models were applied to investigate the relationship between main baseline and clinical characteristics and the probability to have a late resumption of EAdi, as well as the predictors for the use of sedative agents during the first 24 hours (or until EAdi resumption if it occurred before 24 hours). The Box-Tidweel procedure was applied to test the assumption of a linear relationship between continuous independent variables and the logit transformation of the dependent variable. Also, in this case, we applied a stepwise approach in order to determine which specific independent variables made meaningful contributions to the overall prediction. Results were reported as odds ratio with 95% CI. See electronic supplement for more details. For the Cox model examining the relationship between baseline parameters and EAdi resumption during the study period, we used the following variables in the univariate model: Age (year), Sex, APACHE-II (score), BMI, (kg/m2), Mode of ventilation (ref. assisted), PaO2/FiO2 (mmHg), Creatinine (µmol/L), Baseline AKI stage, Baseline bilirubin (µmol/L), Cause of intubation, SAS score during the first 6 hours (score), Use of neuromuscular blocking agents, Use of sedatives, use of propofol, midazolam or fentanyl both as time dependent variable and non-time dependent variables. The dose of sedatives per kg of body weight were also modeled as both time and non-time dependent variables. For the multivariable model we included the presence of sedative infusions used in combination as both time and non-time dependent variables.

For the Cox model examining the relationship between baseline parameters and the probability of EAdi resumption after 24 hours we used the following variables: Age (year), Sex, APACHE-II (score), BMI, (kg/m2), Mode of ventilation (ref. assisted), PaO2/FiO2 (mmHg), Creatinine (µmol/L), Baseline AKI stage, Baseline bilirubin (µmol/L), Cause of intubation, SAS score during the first 6 hours (score), use of neuromuscular blocking agents, use of sedatives, use and doses of propofol, midazolam or fentanyl.

Predictors used in the multivariable models (Logistic or Cox) were detected through the stepwise regression approach that combines forward and backward selection methods in an iterative procedure (significance level of 0.05 both for entry and retention). The validity of the proportional hazards assumption in the Cox regression models was assessed with the test proposed by Harrel and Lee based on Schoenfeld residuals. Concerning logistic models, we observed 35 events (EAdi resumption) and according to the minimal “10 events per variable” rule in medical literature, if anything we were able to evaluate 3 independent predictors in a same logistic model.

EAdi and TFdi: The relationship between EAdi and TFdi was analyzed by a general linear regression model. For patients with measurements available during both controlled and assisted modes of ventilation, both measurements were included in the model. When patients had multiple measurements per ventilation modality available, average EAdi and TFdi values were included to account for differences in repeated measurements between patients. Recordings obtained during reverse triggering were excluded from the analysis. Then, we classified data according to a TFdi value above or below 15% (i.e., TFdi associated with minimal diaphragm contractile activity[12]); differences in EAdi between these groups were assessed with a Mann-Whitney U test. A two-sided p-value less than 0.05 was considered to indicate statistical significance.

All analyses were conducted using SAS version 9.4 (SAS Institute, Cary, NC, USA), R software version 3.3.2 (R Foundation for Statistical Computing, Vienna, Austria) and SPSS version 24.0 (IBM, Corp. USA)

**Table E1.** Characteristics of study population during the study period (n=69), stratified according to time to resumption of EAdi (Early resumption of EAdi = resumption of EAdi by 24 hours; Late resumption of EAdi or catheter disconnection after 24 hs = resumption of EAdi after 24 hours or no recorded resumption).

| **Characteristic** | **Early resumption**  **of EAdi** | **Late resumption of EAdi**  **or catheter disconnection**  **after 24 hs** | **p-value** |
| --- | --- | --- | --- |
| **N** | 35 (50.72) | 34 (49.28) | - |
| **Male, n (%)** | 22 (62.86) | 19 (55.88) | 0.5553 |
| **Age (years)**, mean ± SD | 68.57 ± 15.89 | 57.94 ± 15.73 | 0.0043 |
| **BMI, (kg/m^2^)**, mean ± SD | 29.24 ± 9.27 | 28.10 ± 9.45 | 0.5556 |
| **APACHE-II (score)** |  |  |  |
| Mean ± SD | 25.47 ± 9.28 | 23.76 ± 7.18 | 0.3996 |
| Median (q_1_-q_3_) | 24.50 [18.00-31.00] | 24.50 [19.00-29.00] |  |
| **Reason for ICU admission, n (%)** |  |  | 0.3761 |
| Elective surgery | 0 (0.00) | 1 (2.94) |  |
| Emergency surgery | 3 (8.57) | 2 (5.88) |  |
| Trauma | 1 (2.86) | 4 (11.76) |  |
| Medical | 31 (88.57) | 27 (79.41) |  |
| **Reason for intubation, n (%)** |  |  | 0.3057 |
| Pulmonary | 21 (60.00) | 17 (50.00) |  |
| Hemodynamic | 8 (22.86) | 5 (14.71) |  |
| Neurologic | 4 (11.43) | 10 (29.41) |  |
| Other | 2 (5.71) | 2 (5.88) |  |
| **Intubation time, n (%)** |  |  | 0.9559 |
| Day (6am-9pm) | 29 (82.86) | 28 (82.35) |  |
| Night (9pm-6am) | 6 (17.14) | 6 (17.65) |  |
| **Arterial blood gas**^‡^ |  |  |  |
| FiO_2_, median (q_1_-q_3_) | 0.50 [0.40-0.70] | 0.50 [0.50-0.75] | 0.2185 |
| PaO_2_ (mmHg), mean ± SD | 110.57 ± 30.25 | 132.09 ± 59.74 | 0.2154 |
| PaCO_2_ (mmHg), mean ± SD | 41.70 ± 8.45 | 46.39 ± 12.41 | 0.2024 |
| HCO_3_ (mmol/L)°, mean ± SD | 24.34 ± 6.55 | 22.91 ± 5.98 | 0.3394 |
| PaO_2_ / FiO_2_ (mmHg), mean ± SD | 214.49 ± 78.46 | 237.14 ± 124.33 | 0.3866 |
| **Baseline creatinine (µmol/L), median [IQR]** | 99.00 [72.00-158.00] | 106.50 [70.00-200.00] | 0.9761 |
| **Baseline AKI stage^**^** |  |  | 0.1423 |
| No AKI | 16 (45.71) | 17 (50.00) |  |
| 1 | 15 (42.86) | 7 (20.59) |  |
| 2 | 1 (2.86) | 3 (8.82) |  |
| 3 | 3 (8.57) | 7 (20.59) |  |
| **Baseline bilirubin (µmol/L)†, median [IQR]** | 11.00 [6.00-17.00] | 10.00 [7.00-17.00] | 0.8104 |
| **Ventilatory settings at EAdi catheter connection** |  |  |  |
| Controlled mode, n (%) | 29 (82.86) | 33 (97.06) | 0.1060 |
| PEEP (cmH_2_O), mean ± SD |  |  |  |
| All patients | 7.82 ± 2.51 | 8.29 ± 3.39 | 0.5891 |
| Patients on controlled mode (n=62) | 7.82 ± 2.65 | 8.31 ± 3.44 | 0.7509 |
| Patients on assisted mode (n=7) | 7.84 ± 1.89 | 7.77 ± . | - |
| Mean airway pressure (cmH_2_O), mean ± SD |  |  |  |
| All patients | 12.09 ± 3.17 | 12.93 ± 4.60 | 0.6058 |
| Patients on controlled mode (n=62) | 12.38 ± 3.21 | 13.03 ± 4.63 | 0.7832 |
| Patients on assisted mode (n=7) | 10.68 ± 2.75 | 9.70 ± . | - |
| **Time with available EAdi peak value (hours),** median (q_1_-q_3_) | 46.20 [27.10-67.22] | 95.80 [74.58-118.50] | <.0001 |
| **Tracheostomy during ICU stay, n (%)**^+^ | 4 (11.43) | 3 (8.82) | 1.0000 |
| **Mortality, n (%)** |  |  |  |
| At ICU discharge | 8 (22.86) | 9 (26.47) | 0.7277 |
| At hospital discharge† | 13 (38.24) | 12 (35.29) | 0.8014 |
| **Resumption of EAdi during study period** |  |  |  |
| Subject with complete recordings, n (%) | 35 (100.00) | 26 (76.47) | 0.0022 |
| Time to resumption, n (%) |  |  |  |
| <24 hours | 35 (100.00) | - | - |
| 24 hs – 48 hs | - | 11 (42.31) | - |
| 48 hs – 72 hs | - | 6 (23.08) | - |
| 72 hs – 96 hs | - | 5 (19.23) | - |
| > 96 hours, no resumption | - | 4 (15.38) | - |
| Estimated time (hours) to resumption* |  |  |  |
| Median (q_1_-q_3_) | 0.00 [0.00-7.45] | 53.28 [43.85-84.67] |  |
| Mean ± SE | 3.81 ± 1.06 | 66.99 ± 6.13 | <.0001 |
| Average EAdi (µv) during resumption, mean ± SD | 10.81 ± 8.43 | 6.74 ± 3.42 | 0.0018 |
| **Use of sedative during the first 24 hours, n (%)***^* | 32 (91.43) | 31 (91.18) | 1.0000 |
| Propofol, n (%) | 24 (75.00) | 25 (80.65) | 0.5900 |
| *Cumulative dose (mg/kg), median [q_1_-q_3_]* | 16.67 [6.53-28.70] | 20.95 [10.82-36.30] | 0.1645 |
| Midazolam, n (%) | 25 (78.13) | 21 (67.74) | 0.3533 |
| *Cumulative dose (mg/kg), median [q_1_-q_3_]* | 0.07 [0.03-0.25] | 0.10 [0.05-1.33] | 0.2294 |
| Fentanyl, n (%) | 27 (84.38) | 24 (77.42) | 0.4821 |
| *Cumulative dose (*µ*g/kg), median [q_1_-q_3_]* | 3.19 [1.22-10.70] | 9.64 [2.04-17.51] | 0.1545 |
| **Use of sedatives prior to EAdi resumption (early group) or within the first 24 hours (late group), n (%)** | 10 (28.57) | 31 (91.18) | <.0001 |
| Propofol, n (%) | 10 (100.00) | 25 (80.65) | 0.3072 |
| *Cumulative dose (mg/kg), median [q_1_-q_3_]* | 8.41 [5.71-21.97] | 20.95 [10.82-36.30] | 0.0466 |
| Midazolam, n (%) | 3 (30.00) | 21 (67.74) | 0.0632 |
| *Cumulative dose (mg/kg), median [q_1_-q_3_]* | 0.04 [0.03-0.98] | 0.10 [0.05-1.33] | 0.4069 |
| Fentanyl, n (%) | 8 (80.00) | 24 (77.42) | 1.0000 |
| *Cumulative dose (µg/kg), median [q_1_-q_3_]* | 5.28 [1.88-18.91] | 9.64 [2.04-17.51] | 0.6793 |
| **NMBA use, n (%)^¶^** |  |  | 0.0368 |
| No | 11 (31.43) | 5 (14.71) |  |
| Yes | 24 (68.57) | 29 (85.29) |  |
| Yes, for intubation | 24 (68.57) | 25 (73.53) |  |
| Yes, additional bolus or continuous infusion | 0 (0.00) | 4 (11.76) |  |
| **Sedation analgesia score (during the first 6 hours of EAdi recording)**^#^ |  |  |  |
| Median (q_1_-q_3_) | 2.00 [1.00-3.00] | 1.50 [1.00-2.25] | 0.2707 |

*Abbreviations: APACHE-II: Acute Physiology, Age, Chronic Health Evaluation II; BMI: body mass index; EAdi: electrical activity of the diaphragm; FiO_2_: fraction of inspired oxygen; HCO_3_: bicarbonate; ICU: intensive care unit; NMBA: neuromuscular blocking agents; PaCO_2_: partial pressure of carbon dioxide; PaO_2_: partial pressure of oxygen; PEEP: positive end-expiratory pressure; q_1_: first quartile; q_3_: third quartile; SD: standard deviation; SE: standard error.*
*‡ 63 patients had ABG measured at baseline. ° For 1 patient HCO_3_ was not available.*
*^+^ Tracheostomy performed after 120 hours from first available EAdi.*

*† For 1 patient, data was missing.*

** Estimated time was assessed on the whole study population (69 patients) with Kaplan-Meier approach. Time to resumption is calculated from first available EAdi.*

*^#^Sedation analgesia score not recorded in 54 patients in the first 6 hours.*

**^**^***AKI staging was determined using KDIGO Clinical Practice Guideline for Acute Kidney Injury*

*^6 patients did not receive continuous sedative infusions in the first 24 hours*

^¶^ *Standard doses for neuromuscular blocking agents were used; intubation doses for rocuronium were 0.6-1.2mg/kg, succinylcholine 1-2mg/kg and cisatracurium 0.15-0.2mg/kg. Continuous infusions of rocuronium or cisatracurium were titrated to clinical effect and were dosed as for 0.2 to 0.7 mg/kg/hour and 0.06 to 0.18 mg/kg/hour respectively as per hospital policy.*

**Table E2**. Relationship between baseline parameters and probability to be sedated during the first 24 hours (or until EAdi resumption).

|  | **Odds ratio (95% confidence interval)** | **p-value** | **N** |
| --- | --- | --- | --- |
| **Univariate logistic models** |  |  |  |
| **Age (year)** | 0.933 (0.896-0.971) | 0.0007 | 69 |
| **Sex (ref. Female)** | 0.710 (0.264-1.908) | 0.4971 | 69 |
| **APACHE-II (score)** | 0.965 (0.908-1.024) | 0.2413 | 68 |
| **BMI, (kg/m^2^)** | 0.983 (0.933-1.036) | 0.5247 | 67 |
| **Mode of ventilation (ref. assisted)** | 4.239 (0.760-23.645) | 0.0996 | 69 |
| **PaO_2_/FiO_2_ (mmHg)** | 1.000 (0.996-1.005) | 0.8606 | 63 |
| **Creatinine (µmol/L)** | 1.001 (0.997-1.004) | 0.6882 | 69 |
| **Baseline AKI stage (ref. No AKI)** |  |  | 69 |
| **1** | 0.346 (0.113-1.057) | 0.0625 |  |
| **2** | 1.500 (0.139-16.144) | 0.7380 |  |
| **3** | 1.167 (0.252-5.409) | 0.8439 |  |
| **Baseline bilirubin (µmol/L)** | 1.004 (0.986-1.022) | 0.6760 | 68 |
| **Reason for intubation (ref. Neurologic)** |  |  | 65* |
| **Pulmonary** | 0.494 (0.131-1.857) | 0.7445 |  |
| **Hemodynamic** | 0.343 (0.070-1.684) | 0.2700 |  |
| **Multivariable logistic model** |  |  |  |
| **Age** | 0.933 (0.896-0.971) | 0.0007 | 69 |

*4 patients with other causes of intubation were excluded.

**Table E3**: Estimated time (hours) to resumption for secondary time and voltage thresholds

| 5µV, 12 hours, median (q1-q3) | 18.38 (0.00-53.88) hours |
| --- | --- |
| 5µV, 24 hours, median (q1-q3) | 22.02 (0.00-50.77) hours |
| 7µV, 12 hours, median (q1-q3) | 18.40 (0.00-53.90) hours |

References

1. Goligher EC, Detsky ME, Sklar MC, Campbell VT, Greco P, Amaral ACKB, et al. Rethinking Inspiratory Pressure Augmentation in Spontaneous Breathing Trials. Chest. 2017;151:1399–400.

2. Barwing J, Ambold M, Linden N, Quintel M, Moerer O. Evaluation of the catheter positioning for neurally adjusted ventilatory assist. Intensive Care Med. 2009;35:1809–14.

3. Goligher EC, Fan E, Herridge MS, Murray A, Vorona S, Brace D, et al. Evolution of Diaphragm Thickness during Mechanical Ventilation. Impact of Inspiratory Effort. Am J Respir Crit Care Med. 2015;192:1080–8.

4. Piquilloud L, Beloncle F, Richard J-CM, Mancebo J, Mercat A, Brochard L. Information conveyed by electrical diaphragmatic activity during unstressed, stressed and assisted spontaneous breathing: a physiological study. Ann Intensive Care. 2019;9:89.

5. Carteaux G, Córdoba-Izquierdo A, Lyazidi A, Heunks L, Thille AW, Brochard L. Comparison Between Neurally Adjusted Ventilatory Assist and Pressure Support Ventilation Levels in Terms of Respiratory Effort. Crit Care Med. 2016;44:503–11.

6. Beck J, Emeriaud G, Liu Y, Sinderby C. Neurally-adjusted ventilatory assist (NAVA) in children: a systematic review. Minerva Anestesiol. 2016;82:874–83.

7. Section 2: AKI Definition. Kidney Int Suppl. 2012;2:19–36.

8. Riker RR, Picard JT, Fraser GL. Prospective evaluation of the Sedation-Agitation Scale for adult critically ill patients. Crit Care Med. 1999;27:1325–9.

9. Akoumianaki E, Lyazidi A, Rey N, Matamis D, Perez-Martinez N, Giraud R, et al. Mechanical Ventilation-Induced Reverse-Triggered Breaths: A Frequently Unrecognized Form of Neuromechanical Coupling. Chest. 2013;143:927–38.

10. Grambsch PM, Therneau TM. Proportional hazards tests and diagnostics based on weighted residuals. Biometrika. Oxford Academic; 1994;81:515–26.

11. Austin PC. Statistical power to detect violation of the proportional hazards assumption when using the Cox regression model. J Stat Comput Simul. 2018;88:533–52.

12. Goligher EC, Dres M, Fan E, Rubenfeld GD, Scales DC, Herridge MS, et al. Mechanical Ventilation–induced Diaphragm Atrophy Strongly Impacts Clinical Outcomes. Am J Respir Crit Care Med. 2017;197:204–13.
