# Supplementary figures and images for: Duration of diaphragmatic inactivity after endotracheal intubation of critically ill patients
Source: Crit Care. 2021 Jan 11;25:26. doi: 10.1186/s13054-020-03435-y (PMC7798017; doi:10.1186/s13054-020-03435-y)

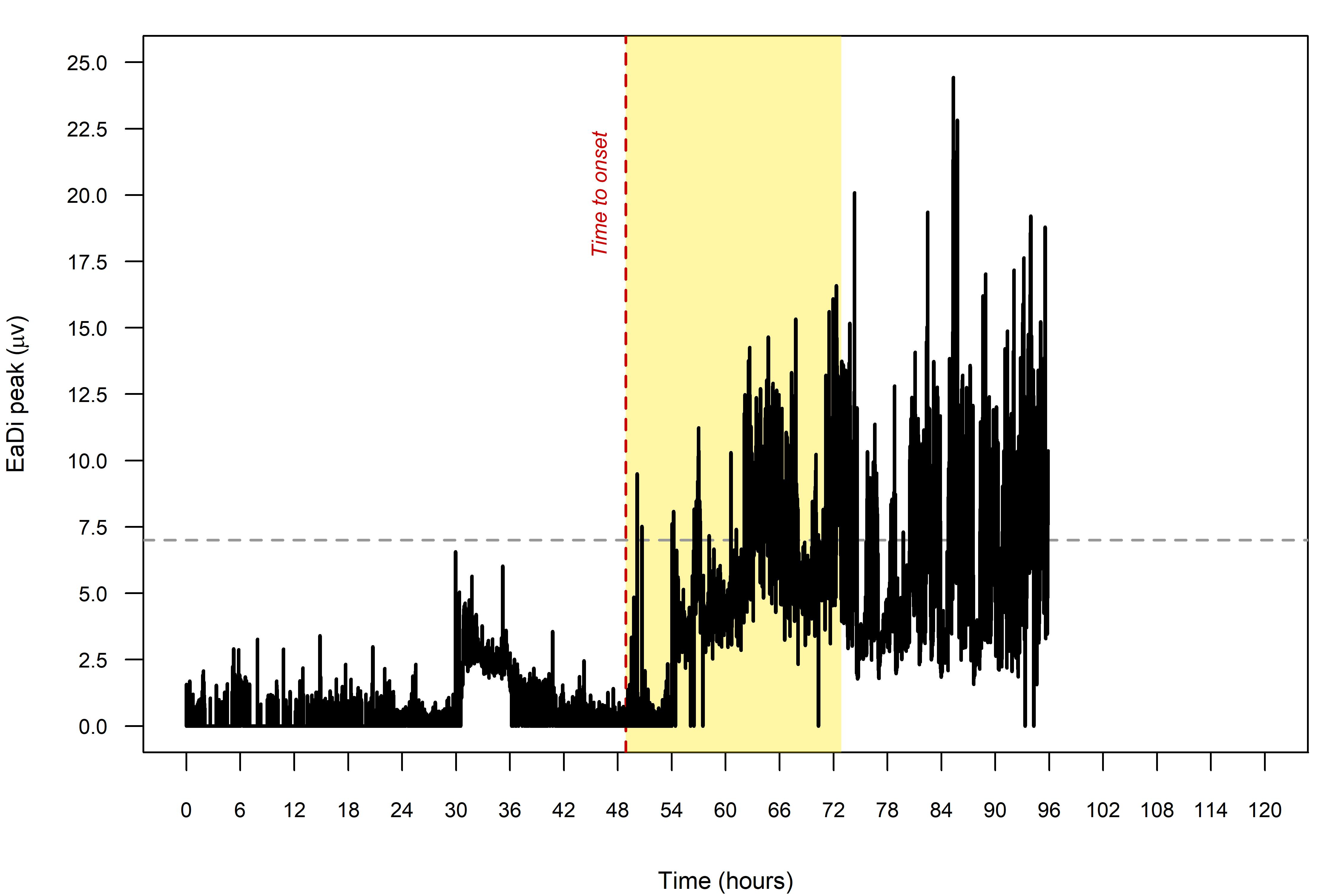

Supplement: Supplementary file 2 — Additional file 2. Figure E1. [file 13054_2020_3435_MOESM2_ESM.tif]

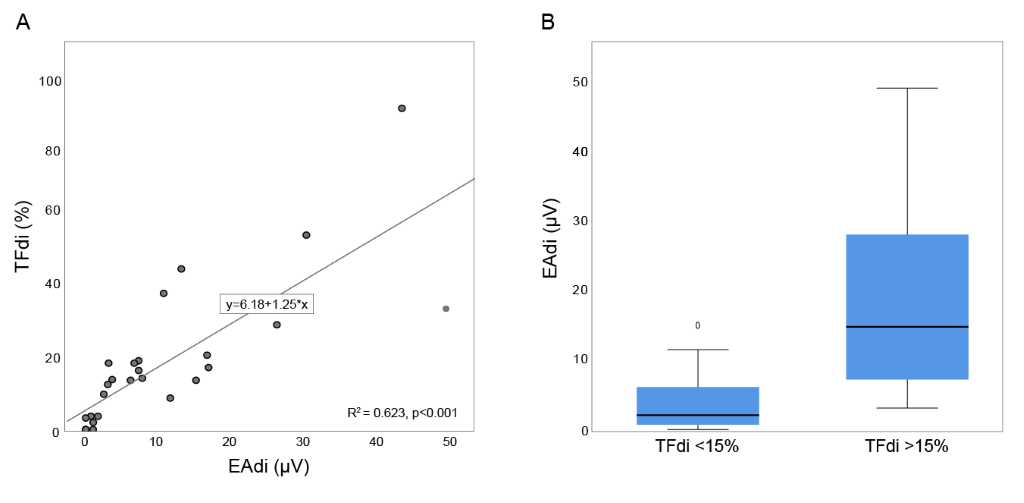

Supplement: Supplementary file 3 — Additional file 3. Figure E2. [file 13054_2020_3435_MOESM3_ESM.png]

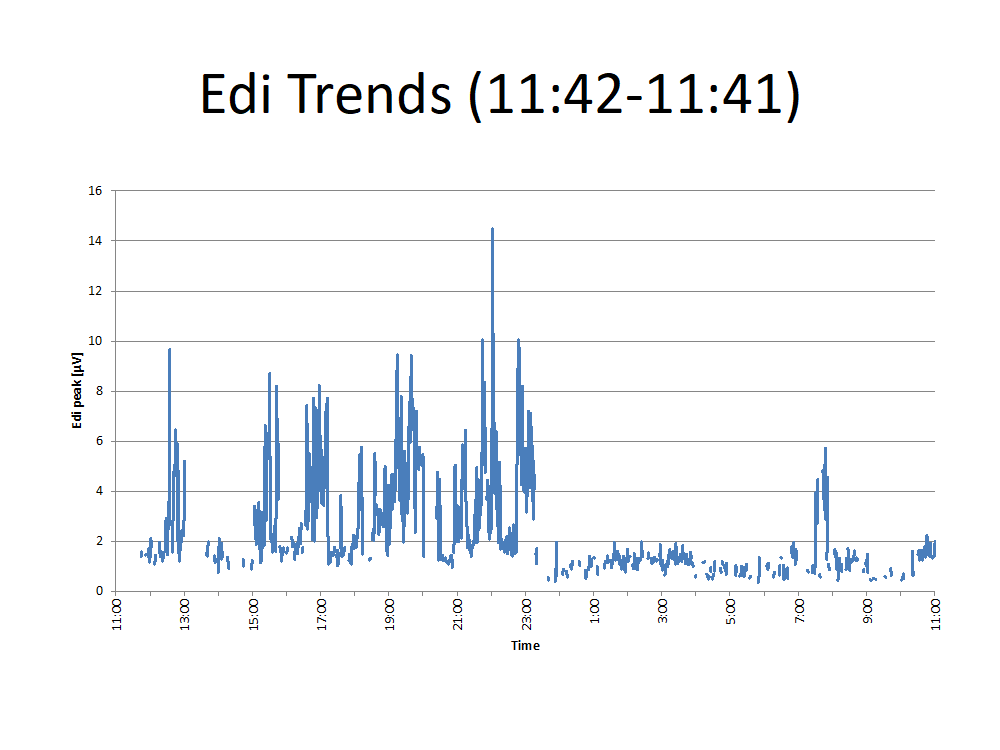

Supplement: Supplementary file 4 — Additional file 4. Figure E3. [file 13054_2020_3435_MOESM4_ESM.png]
